# Coping behaviors and depressive status in individuals with type 2 diabetes mellitus

**DOI:** 10.1186/s12991-019-0235-5

**Published:** 2019-07-17

**Authors:** Norio Yasui-Furukori, Hiroshi Murakami, Hideyuki Otaka, Hirofumi Nakayama, Masaya Murabayashi, Satoru Mizushiri, Koki Matsumura, Jutaro Tanabe, Yuki Matsuhashi, Miyuki Yanagimachi, Kazuhiko Nakamura, Makoto Daimon, Norio Sugawara

**Affiliations:** 1 Department of Neuropsychiatry, Hirosaki University Graduate School of Medicine, Hirosaki, Japan; 2 Department of Endocrinology and Metabolism, Hirosaki University Graduate School of Medicine, Hirosaki, Japan; 3 Department of Clinical Epidemiology, Translational Medical Center, National Center of Neurology and Psychiatry, Kodaira, Tokyo Japan; 4 Department of Psychiatry, Dokkyo Medical University School of Medicine, Mibu, Shimotsuga, Tochigi Japan

**Keywords:** Cross-sectional studies, Coping behaviors, Depressive symptoms, Japanese, Type 2 diabetes

## Abstract

**Objective:**

Type 2 diabetes mellitus (T2DM) is associated with a high prevalence of depression, which is influenced by personality traits and coping style. However, these psychological factors have not been well studied in individuals with T2DM. The association between coping behaviors and the reported levels of depressive symptoms was examined in individuals with T2DM.

**Methods:**

The subjects were 435 T2DM patients (mean age 63.1 ± 12.6 years). Depressive status, personality traits and coping behaviors were assessed using the Center for Epidemiologic Studies Depression Scale (CES-D) and the Brief Scale for Coping Profile (BSCP). Lifestyle factors and glycated hemoglobin A1c (HbA1c) levels in the patients were also included in the analyses.

**Results:**

Among the 435 subjects with T2DM, 130 (29.9%) exhibited possible depression, and 68 (15.6%) displayed probable depression. After adjustment for confounders, logistic and multiple regression analyses revealed that certain coping profile scores, such as Changing one’s point of view, Emotional expression involving others and Avoidance and suppression, were consistently and significantly associated with the presence and severity of depression. No relationship was found between depression and HbA1c.

**Conclusion:**

These findings indicate that Maladaptive emotion-focused coping strategies, such as Emotional expression involving others and Avoidance and suppression, are protective factors and that Adaptive emotion-focused coping, such as Changing one’s point of view, is a risk factor for depression in T2DM patients. Psychological intervention focusing on the coping profile may reduce depressive symptoms. Additional studies are needed to examine the relationships between psychological factors and depressive symptoms using a longitudinal study design.

## Introduction

The high prevalence of type 2 diabetes mellitus (T2DM) as a lifestyle-related disease has become a burden, and T2DM can increase the risk of both serious physical and mental health issues. Depression is twice as common in patients with diabetes compared with the general population, with differing rates between patients with type 1 diabetes (21.3%) and T2DM (27.0%) [1]. A recent meta-analysis suggested that the pooled relative risk for depression is 1.41–1.43 in patients with T2DM [2, 3].

Patients with diabetes have a variety of emotional reactions, such as shame, fear, shock and guilt [4]. Notably, insulin therapy in particular may be associated with increased psychological distress [5]. Emotional problems such as depression or diabetes-specific distress might complicate the required self-management of the disease [6] and limit the persons’ management of self-care activities (exercise, diet, rest) necessary to achieve adequate glycemic control [7–10].

A recent review reported that psychotherapeutic interventions (some of which were combined with diabetes education) had a moderate-to-large effect on depressive symptoms and a moderate-to-large effect on glycemic control. On the other hand, pharmacological treatment with selective serotonin reuptake inhibitor (SSRI) also had a moderate-to-large effect on depressive disorders but less of an effect on glycemic control. Therefore, the development of psychological intervention is important for the management of glycemic control.

The coping profile is a mediator that influences mood. Coping has been described as a process in which cognitive or behavioral strategies are developed to manage specific internal and/or external sources of psychological stress [11, 12]. The coping strategies used to address diabetes can play a key role in the maintenance and duration of the disease as well as the psychosocial adjustment to diabetes [13–15]. However, few studies have examined the potential link between coping behaviors and depressive symptoms among individuals with T2DM.

In the present study, we aimed to investigate the prevalence of depressive symptoms and to assess the relationship between coping behaviors and depressive symptoms among individuals with T2DM. We hypothesized that dysfunctional coping behaviors, such as Maladaptive emotion-focused coping strategies, would be associated with depressive symptoms.

## Methods

### Participants

This study was approved by the ethics committee of the Graduate School of Medicine, Hirosaki University, and written informed consent was obtained from each subject before participation in this study. A total of 728 individuals with T2DM who received treatment for at least 1 year at the Department of Endocrinology and Metabolism at the Hirosaki University Hospital were enrolled. We recruited 945 patients, and 728 agreed to take part in the study. The remaining 217 patients were excluded because of a decline (41 patients), moderate-to-severe dementia (85 patients), blindness (23 patients), or moderate-to-severe psychiatric diseases (e.g., schizophrenia and bipolar disorder; 68 patients).

A total of 611 out of 728 patients returned the questionnaires and were regarded as participants with informed consent, and the average (standard deviation) age, body mass index (BMI), CES-D and HbA1c were 63.5 (12.8) years, 24.9 (4.3), 13.3 (8.4) and 7.1 (0.9)%, respectively. Ultimately, 435 (59.7%) patients had complete questionnaires and were included as subjects in this study. No differences in demographic data were found between completers (*n* = 435) and noncompleters (*n* = 176). Routine glycated hemoglobin A1c (HbA1c) analysis was performed at least four times per year for these patients. Among the subjects, 385 were receiving an oral hypoglycemic agent, and 215 were receiving insulin therapy. The demographic data (age, sex, alcohol consumption, smoking, marital status, solitary living, and exercise habits) and medical histories of the patients were obtained from questionnaires and medical records.

The Japanese version of the Center for Epidemiologic Studies Depression Scale (CES-D) was administered to all of the participants to measure their depressive symptoms [16]. The CES-D is a 20-item self-reported measure that focuses on the depressive symptoms the patient experienced during the week prior to completing the questionnaire. The maximum score is 60, with higher scores indicating more severe depressive symptoms. The standard cutoff score of − 16 for the CES-D had excellent sensitivity (91.3%) but low specificity (60.8%). A cutoff score of − 21 on the CES-D yielded an optimal balance between sensitivity (78.3%) and specificity (74.3%), with a positive predictive value (PPV) of 48.6% and a negative predictive value (NPV) of 91.7% [17]. In addition, our previous study indicated that the stratum-specific likelihood ratios (SSLRs) were 0.13 (95% CI 0.04–0.40), 3.68 (95% CI 1.37–9.89), and 24.77 (95% CI 14.97–40.98) for CES-D scores of 0–16, 17–20, and above 21, respectively [18]. Therefore, based on our previous study, a reported CES-D score of − 16 indicated possible depression, while a reported CES-D score of − 21 was indicative of probable depression.

The Brief Scale for Coping Profile (BSCP) was used to assess coping behaviors in the study. The BSCP consists of 18 items rated on a 4-point Likert scale [19–21]. We asked patients to check a box indicating the frequency at which they used the strategy described in a particular item in stressful situations using a scale from 1 (almost never) to 4 (very often). The scale assesses an individual’s ability to cope with stressful daily circumstances using six subscales: “Active solution (AC)”, “Seeking help for a solution (S)”, “Changing mood (CM)”, “Changing one’s point of view (CV)”, “Avoidance and suppression (AV)”and “Emotional expression involving others (EE)”. These six subscales reflect three different coping dimensions: problem-focused (AC and S), Adaptive emotion-focused (CM and CV), and Maladaptive emotion-focused (AV and EE) coping strategies. A high score on a certain subscale indicates that the respondent frequently selected that coping method.

### Statistical analysis

In the present study, comparisons of several factors between patients with and without depression were performed using *t*-tests and Chi-square tests. The data are presented as the means ± standard deviations. A *p*-value < 0.05 indicated statistical significance. The factors associated with depression were examined using logistic regression analyses with forced entry; they included age, sex, BMI, HbA1c, presence/absence of smoking, habitual alcohol consumption, living alone, exercise habits, and insulin use, as well as each score for the 6 coping profiles or 3 coping dimensions. In addition, linear regression analyses with forced entry were performed to examine the correlation between the severity of depression (total CES-D scores) and several factors, including 3 coping dimensions. The dummy variables included were as follows: male = 1, female = 2, living with family = 0, living alone = 1, presence of spouse = 1, absence of spouse = 2, presence of smoking = 1, absence of smoking = 2, presence of alcohol consumption = 1, absence of alcohol consumption = 2, no exercise = 1, exercise once a week = 2, exercise 2–3 days per week = 3, exercise 4–5 days per week = 4, exercise almost every day = 5 and insulin nonuse = 0, insulin use = 1. A *p*-value < 0.05 indicated statistical significance. SPSS statistical software for Windows, version 25.0, was used for all analyses. A *p* < 0.0025 and a *p* < 0.0033 were regarded as significant using Bonferroni’s correction due to multiple testing in comparison between patients with and without depression and simple correlations, respectively.

## Results

Of the 435 patients, 130 (29.9%) had possible depression based on the standard CES-D cutoff value of 15.5. However, we designated 21.5 as the solid CES-D cutoff value, as previously described, and defined patients with CES-D scores of 21 and higher as showing depression. Thus, 64 (14.7%) patients exhibited probable depression.

Significant differences in BMI, smoking habits, Changing one’s point of view, Emotional expression involving others, and Avoidance and suppression were found between the patients with and without depression, but no differences in age, HbA1c, major lifestyle factors or marital status were observed (Table 1).

**Table 1:** Characteristics of subjects with and without depression

	No depression (*n *= 367)	Depression (*n *= 68)	Significant
Sex			
Male (*n*, %)	220 (60%)	36 (53%)	ns
Female (*n*, %)	147 (40%)	32 (47%)	
Age (yo)	63.6 ± 12.7	60.3 ± 12.3	*p *= 0.043
BMI	24.9 ± 4.1	26.2 ± 5.0	
HbA1c (%)	7.1 ± 0.9	7.1 ± 0.9	ns
Insulin			
User (*n*, %)	185 (50%)	30 (44%)	ns
Non user (*n*, %)	182 (50%)	38 (54%)	
Smoking habit			
Yes (*n*, %)	51 (14%)	21 (31%)	*p *= 0.018
No (*n*, %)	316 (86%)	47 (69%)	
Habitual alcohol consumption			
Yes (*n*, %)	129 (35%)	18 (26%)	ns
No (*n*, %)	238 (65%)	50 (74%)	
Exercise frequency			
None (*n*, %)	186 (51%)	41 (60%)	ns
Once a week (*n*, %)	28 (8%)	4 (6%)	
2–3 times a week (*n*, %)	54 (15%)	10 (15%)	
4–5 times a week (*n*, %)	35 (10%)	5 (7%)	
Almost everyday (*n*, %)	64 (17%)	8 (12%)	
Single			
Yes (*n*, %)	105 (28%)	27 (40%)	ns
No (*n*, %)	262 (72%)	41(60%)	
Living alone			
Yes (*n*, %)	44 (12%)	10 (15%)	ns
No (*n*, %)	323 (88%)	58 (85%)	
CES-D	10.4 ± 5.1	27.9 ± 7.2	*p = 0.000*
Coping profiles			
Active solution	6.2 ± 2.6	6.7 ± 2.7	ns
Seeking help for a solution	8.0 ± 2.8	8.4 ± 2.7	ns
Changing mood	8.1 ± 2.6	8.0 ± 2.7	ns
Changing one’s point of view	7.5 ± 2.4	8.3 ± 2.3	*p *= 0.008
Avoidance and suppression	9.6 ± 2.0	8.4 ± 2.4	*p = 0.000*
Emotional expression involving others	11.3 ± 1.3	10.2 ± 2.3	*p = 0.000*
Coping dimensions			
Problem focused	14.2 ± 4.8	15.1 ± 4.7	ns
Adaptive emotion focused	15.6 ± 4.1	16.4 ± 3.9	ns
Maladaptive emotion focused	20.9 ± 2.7	18.6 ± 4.2	*p = 0.000*

Data are shown as mean ± SD

*p *< 0.0025 was regarded as significant using Bonferroni’s correction due to multiple testing (italic)

The logistic regression analysis that included patients with depression revealed associations with smoking habits, alcohol consumption habits, and Avoidance and suppression (Table 2). Multiple regression analysis indicated that BMI, smoking habits, alcohol consumption habits, marital status, Changing one’s point of view, Emotional expression involving others and Avoidance and suppression were correlated with the severity of depression as measured by the total CES-D score (Table 3).

**Table 2:** Logistic regression results for factors associated with depression among type 2 DM patients

	Exp(B)	95% CI
Sex	1.19	0.64–2.2
Age	1.00	0.98–1.03
BMI	1.05	0.98–1.13
HBA1c	1.07	0.76–1.53
Smoking	0.32	0.15–0.67**
Alcohol	1.74	0.88–3.46
Exercise frequency	1.00	0.82–1.22
Single	1.57	0.76–3.26
Living alone	1.21	0.46–3.20
Coping profiles		
Active solution	1.02	0.89–1.17
Seeking help for a solution	1.07	0.93–1.22
Changing mood	0.99	0.87–1.12
Changing one’s point of view	1.29	1.11–1.49**
Avoidance and suppression	0.76	0.64–0.89**
Emotional expression involving others	0.76	0.63–0.91**
Sex	1.18	0.66–2.17
Age	1	1.00–1.04
BMI	1.00	0.98–1.03
HBA1c	1.05	0.74–1.48
Smoking	0.34	0.17–0.70**
Alcohol	1.70	0.86–3.37
Exercise frequency	1.01	0.83–1.22
Single	1.58	0.78–3.21
Living alone	1.15	0.44–3.00
Coping dimensions		
Problem focused	1.04	0.97–1.11
Adaptive emotion focused	1.12	1.02–1.22*
Maladaptive emotion focused	0.77	0.70–0.84***

Logistic regression analyses for coping profiles and coping dimensions were separately performed including confounding factors such as sex, age, BMI, presence/absence of smoking, habitual alcohol consumption, living alone, and exercise habits, HbA1c, and insulin use/no use

* *p* < 0.05, ** *p* < 0.01, *** *p* < 0.001

**Table 3:** Simple and multiple regression results for factors associated with severity of depression among type 2 DM patients

	*r*	Significance	Beta	Significance
Sex	0.044	0.181	0.042	0.374
Age	− 0.030	0.265	0.061	0.234
BMI	0.035	0.234	0.02	0.674
HBA1c	− 0.001	0.490	− 0.015	0.739
Smoking	− 0.083	0.043	− 0.120	*0.008*
Alcohol	0.068	0.078	0.072	0.121
Exercise frequency	− 0.045	0.176	− 0.017	0.704
Single	0.116	0.008	0.114	*0.027*
Living alone	0.053	0.134	− 0.006	0.911
Coping profiles				
Active solution	0.151	*0.001*	0.046	0.403
Seeking help for a solution	0.137	*0.002*	0.103	0.064
Changing mood	− 0.003	0.471	− 0.013	0.791
Changing one’s point of view	0.129	*0.004*	0.175	*< 0.001*
Avoidance and suppression	− 0.332	*< 0.001*	− 0.275	*< 0.001*
Emotional expression involving others	− 0.325	*< 0.001*	− 0.226	*< 0.001*
Multiple correlation coefficients			0.487	*< 0.001*
Sex	0.044	0.181	0.033	0.485
Age	− 0.030	0.265	0.052	0.313
BMI	0.035	0.234	0.033	0.497
HBA1c	− 0.001	0.490	− 0.020	0.674
Smoking	− 0.083	0.043	− 0.116	*0.011*
Alcohol	0.068	0.078	0.071	0.127
Exercise frequency	− 0.045	0.176	− 0.018	0.693
Single	0.116	0.008	0.113	*0.028*
Living alone	0.053	0.134	− 0.008	0.882
Coping dimensions				
Problem focused	0.163	*< 0.001*	0.135	*0.007*
Adaptive emotion focused	0.074	0.063	0.131	*0.011*
Maladaptive emotion focused	− 0.384	*< 0.001*	− 0.429	*< 0.001*
Multiple correlation coefficients			0.525	*< 0.001*

Linear regression analyses for coping profiles and coping dimension were separately performed including confounding factors such as sex, age, BMI, presence/absence of smoking, habitual alcohol consumption, living alone, and exercise habits, HbA1c, and insulin use/no use

Italic show statistically significant

*p* < 0.003 was regarded as significant using Bonferroni’s correction due to multiple testing in simple correlations

## Discussion

The results of this study indicated that Avoidance and suppression, and Emotional expression involving others were inversely associated with depression and that Changing one’s point of view was significantly associated with depression in logistic regression analyses and linear regression analysis after adjustment for confounders. Among the coping dimension, Adaptive emotion-focused coping was negatively correlated with depression, and Maladaptive emotion-focused coping was significantly correlated with depression. In addition, depression was inversely associated with problem-focused coping dimensions such as Active solution or Seeking help for a solution in multiple regression analyses. This study is the first to investigate the relationship between coping profiles/dimensions and depression in T2DM. These findings indicate that Avoidance and suppression, and Emotional expression involving others, termed Maladaptive emotion-focused coping, are protective factors. Changing one’s point of view, termed Adaptive emotion-focused coping, is a risk factor for depression in T2DM patients. These findings were contradictory to our expectations that a functional coping style is considered protective, while a nonfunctional coping style is considered as a risk factor for depression.

In fact, a cross-sectional study [22] and longitudinal study [23] in Western populations consistently demonstrated that task-oriented coping was protective against depression, whereas emotional-oriented coping was a risk factor for depression. We cannot clearly explain this discrepancy, although one possible explanation could be the difference in not only demographics but also culture between Western and Asian countries. The participants in our sample were older and had more severe disease than those in previous studies [22, 23].

The differences between the results of this study and those of previous studies regarding the effect of coping strategy on depression may be indicative of cultural differences in how patients from various cultures distract or vent their diabetes-related distress [24–26]. In the Aomori region of Japan where this study was conducted, there are shaman called ITAKO or KAMISAMA who make predictions, tell fortunes and provide medical care through their spiritual or religious power [27]. Out of 670 participants, 232 (34.6%) had experience consulting a shaman. Females had an increased tendency to consult shamans, and they went to shamans to address personal and family illness. Only 20% of the participants who visited shamans experienced no change, whereas the remaining participants felt healed. Thus, the subject may not have the stamina that is associated with protection against depression [28] but may have helplessness/hopelessness, which is a risk factor for depression [28]. On the other hand, a Nigerian study showed that high religiosity is associated with positive coping skills [28]. The discrepancy with previous studies may be explained by such spiritual or religious backgrounds [28]. Several studies with various patient groups suggest that increased spiritual or religious coping decreases anxiety, hopelessness or depression in T2DM patients, and it stimulates psychological functions or quality of life [29–31]. Therefore, religiosity associated with coping style should be assessed in Japan, particularly in the Aomori region.

Similar to Avoidance and suppression, Denial is used in ‘an attempt to reject the reality of the stressful event’ [32]. Denial is transiently effective in anxiety and/or depression, although it is thought to deteriorate anxiety and/or depression in the long term. However, patients with severe chronic disease may give up active coping gradually with the progression of diabetes-related conditions such as amputation or dialysis.

In the present study, the prevalence of possible and probable untreated depression among individuals with T2DM was 29.9% and 15.6%, respectively. The prevalence of depression in our study was within the range of previous results (15–43%) [33–36]. Untreated depression is associated with a higher number and increased the severity of physical and mental health comorbidities [37]. Notably, the prevalence of depression in the study population was high compared to that in the general population in the same area. Depression is frequently combined with sleep problems [36], anxiety [38], and cardiovascular disease [39]. Moreover, depression is often associated with risky health behaviors, such as poor diet, sedentariness and smoking, which result in obesity and diabetes [40]. Therefore, the early detection of potential depression in individuals with T2DM is clinically important.

Our previous study, which included the same sample population as this study, demonstrated that an emotion-oriented coping style was associated with insomnia, a symptom of depression, as measured using the Pittsburgh Sleep Quality Index [41]. Avoidance and suppression was a protective factor not only for depression in the current study but also for insomnia in the previous study.

This study has several notable limitations. First, the assessment of depressive symptoms was based on only the CES-D rather than clinician-administered structured diagnostic interviews based on established criteria, such as the Diagnostic and Statistical Manual of Mental Disorders, Fourth Edition (DSM-IV). The second limitation of this study was the recruitment strategy, which involved the recruitment of individuals with T2DM from the clinical setting of only one institute and excluded patients with severe mental illness, including major depressive disorders. The untreated individuals enrolled in the current study may not be representative of patients with clinical depression, and the severity of depressive symptoms among our participants might be lower than that among clinical T2DM patients. Third, data on several potential confounding factors, such as life events, economics, traumatic experiences and complications associated with T2DM, were not obtained because of strict ethical considerations and a reluctance to share medical information. This limitation is important because interpersonal relationships among family members and the severity of T2DM complications may have influenced the results of this study. In addition, 40% of the patients who responded to the questionnaire were excluded from the analysis. Because the sample comprised elderly patients, which suggests mild cognitive dysfunction among the participants, completing several kinds of questionnaires might be difficult. Therefore, the bias cannot be excluded entirely. Finally, this study is limited by its cross-sectional design; thus, we could not determine a causal relationship between coping behaviors/personality and the onset of depressive symptoms among the patients in our study population. A follow-up survey is needed to investigate these associations.

## Conclusions

Avoidance and suppression, Emotional expression involving others and Changing one’s point of view were significantly associated with depressive symptoms. These findings indicate that coping behaviors may affect depressive symptoms among individuals with T2DM. Furthermore, T2DM patients might benefit from psycho-educational interventions, such as supportive psychotherapy; however, cognitive behavioral therapy, which is designed to help patients change their point of view and reduce their Avoidance and suppression behaviors when coping with the symptoms of their illness, may be harmful. Additional studies using a longitudinal study design are needed to examine the relationships between coping behaviors and depressive symptoms among T2DM patients.

## Data Availability

Not applicable.
